# Achieving efficient almost CO-free hydrogen production from methanol steam reforming on Cu modified α-MoC†

**DOI:** 10.1039/d3ra07448j

**Published:** 2024-01-10

**Authors:** Wen Jiang, Aonan Liu, Ming Yao, Yuchun Zhang, Peng Fu

**Affiliations:** a School of Agricultural Engineering and Food Science, Shandong University of Technology Zibo 255000 China zhangyc@sdut.edu.cn fupengsdut@163.com

## Abstract

Methanol, serving as a hydrogen carrier, is utilized for hydrogen production through steam reforming, a promising technology for on-vehicle hydrogen applications. Despite the impressive performance of noble-metal catalysts in hydrogen generation, the development of highly efficient non-noble-metal heterogeneous catalysts remains a formidable challenge. In our investigation, we systematically controlled the influence of the MoC phase on the dispersion of active copper metal to enhance the catalytic performance of methanol steam reforming (MSR). Within the Cu/MoC catalyst systems, featuring MoC phases including α-MoC_1−*x*_ and Mo_2_C phases, alongside MoO_2_ phases, the Cu/α-MoC catalyst exhibited exceptional catalytic efficacy at 350 °C. It achieved a remarkable hydrogen selectivity of up to 80% and an outstanding CO selectivity of 0. Notably, its hydrogen production rate reached 44.07 mmol g_cat_^−1^ h^−1^, surpassing that of Cu/Mo_2_C (37.05 mmol g_cat_^−1^ h^−1^), Cu/MoO_2_ (19.02 mmol g_cat_^−1^ h^−1^), and commercial CuZnAl (38 mmol g_cat_^−1^ h^−1^) catalysts. Additionally, we introduced the concept of the (Cu_1_–Cu_*n*_)/α-MoC catalyst, wherein Cu atoms are immobilized on the α-MoC surface, facilitating the coexistence of isolated Cu atoms (Cu_1_) and subnanometer copper cluster (Cu_*n*_) species at a high dispersibility. This innovative design capitalizes on the robust interaction between the α-MoC_1−*x*_ phase and the Cu active center, yielding a substantial augmentation in the catalytic activity.

## Abstract 2

Methanol, serving as a hydrogen carrier, is utilized for hydrogen production through steam reforming, a promising technology for on-vehicle hydrogen applications.

## Graphical abstract

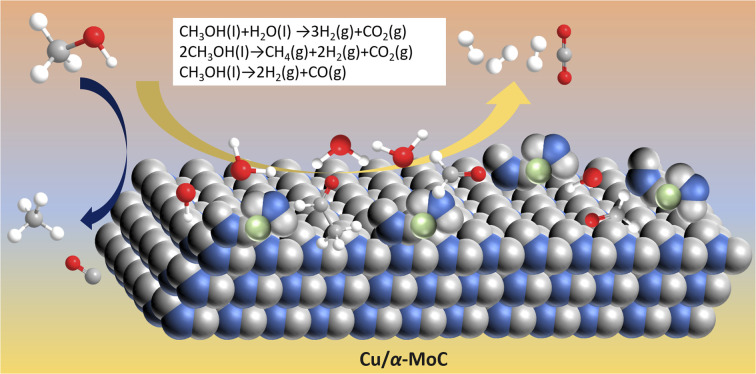

## 1 Introduction

In recent decades, the depletion of fossil energy resources and the escalating environmental pollution crisis have reached alarming proportions. The expansion of renewable energy sources is instrumental in advancing the objectives of attaining carbon neutrality.^1,2^ Consequently, the exploration of alternatives to fossil fuels has emerged as a prominent and pressing research endeavor.^3^ Among these alternatives, proton exchange membrane fuel cells (PEMFCs) have garnered significant attention due to their attributes of high efficiency, exceptional power density, and environmental friendliness.^4,5^ Notably, PEMFCs operate without the thermodynamic constraints imposed by the Carnot cycle, enabling more effective energy extraction from fuel sources.^6,7^ Methanol, characterized by its high hydrogen–carbon ratio and low energy density, stands out as a promising candidate. Methanol is industrially synthesized through the hydrogenation of syngas derived from natural gas or coal. Additionally, it can be produced by the hydrogenation of CO_2_.^8^ Employing methanol as a medium for both hydrogen and carbon, its function as a carrier for renewable energy holds the potential to significantly contribute to the realization of carbon neutrality.^9^ Notably, the reforming of methanol does not entail the cleavage of C–C bonds, and the reforming reactions occur at relatively low temperatures (250–350 °C).^10^ Hence, methanol has emerged as a leading liquid fuel for hydrogen production. Given the inherent safety challenges associated with hydrogen storage and transportation, MSR has gained widespread recognition across various industries as a potential solution.^11–13^

It is noteworthy that MSR reactions employ copper-based catalysts. The commercially available copper-based catalyst CuO/ZnO/Al_2_O_3_, used extensively in MSR, exhibits remarkable catalytic activity and selectivity.^14^ However, existing literature highlights the susceptibility of copper-based catalysts to deactivation, with their performance heavily influenced by the copper state. Deactivation primarily arises from changes in the valence states of active metals, coke deposition, or the occurrence of hot sintering processes.^15–17^

As research and development endeavors in the field of MSR catalysts continue to evolve, there arises an urgent demand for the development of novel and efficient catalysts. Molybdenum carbide-based catalysts have demonstrated exceptional catalytic performance across a range of reaction systems, including photocatalytic hydrogen evolution, water electrolysis, and MSR.^18–20^ Consequently, various metals, such as Ni, Pd, and Pt, have been investigated as promoters or carriers for molybdenum carbide-based catalysts.^20–23^ For instance, Cai *et al.*^22^ utilized a programmed heating approach to load varying quantities of Pd onto α-MoC; notably, 0.5 wt% Pd/MoC displayed an impressive average turnover frequency of up to 807 mol_H_2__ mol_Pd_^−1^ h^−1^. Even after continuous operation for 360 hours at 240 °C, this catalyst exhibited stable catalytic activity, attributable to the strong interaction between Pd atoms and the α-MoC_1−*x*_ phase. Similarly, Lin *et al.*^20^ reported that Pt/MoC catalysts displayed outstanding catalytic activity and stability due to synergistic effects between Pt and α-MoC, as well as the dual functionality of Pt/α-MoC. During hydrolysis, α-MoC provided highly active sites, generating hydroxyl groups and thereby enhancing the hydrogen production activity of Pt/α-MoC.

In response to the prevalence of precious-metal-loaded molybdenum carbide-based catalysts, this study embarked on the synthesis of a series of Cu/MoC catalysts using diverse preparation methodologies. These catalysts were subsequently assessed for their performance in the MSR process, coupled with hydrogen generation. The distinctive characteristics of the Cu/MoC catalysts were meticulously examined to shed light on the constructive interplay between the MoC phase and the active Cu centers. Furthermore, the study delved into the influence of reaction variables, including reaction temperature and copper loading. Ultimately, an in-depth analysis was conducted to evaluate the stability and elucidate the reaction pathway of these MSR catalysts.

## 2 Materials and methods

### 2.1 Catalyst preparation

The MoO_3_-p1 support was prepared as follows: ammonium molybdate ((NH_4_)_6_Mo_7_O_24_·4H_2_O) was procured from Tianjin Chemical Reagent Kaida Chemical Factory. To create the MoO_3_-p1 support, a precise quantity of ammonium molybdate was dissolved in ultrapure water with the aid of ultrasonic treatment. The pH of the solution was adjusted to 3.7, followed by a 2 hours sonication process to ensure thorough mixing and homogeneity. Subsequently, the solvent was evaporated using a water bath, leaving behind a solid residue. This solid was then vacuum-dried overnight at 60 °C to eliminate any residual moisture and achieve complete solidification. The resulting precipitate was further ground into a fine powder to enhance its surface area and reactivity. Finally, the powder underwent calcination in air at 500 °C for 4 hours, resulting in the formation of the MoO_3_-p1 precursor, a versatile support material suitable for various applications.^20^ The catalyst preparation process is illustrated in Fig. S1.†

The MoO_3_-p2 support was prepared as follows: ammonium molybdate powder was subjected to calcination in a muffle furnace with a heating rate of 10 °C min^−1^ until reaching 500 °C. The resulting calcined material was subsequently ground to achieve a particle size of 40–50 mesh, yielding the MoO_3_-p2 precursor.

α-MoC catalyst was prepared as follows: the α-MoC catalyst was synthesized using the ammonia pre-purification method. The MoO_3_-p2 precursor was loaded into a fixed-bed quartz tube reactor. The powder was gradually heated at a rate of 5 °C min^−1^ while pre-purified NH_3_ gas (100 mL min^−1^) was passed through the reactor. The temperature was incrementally increased to 700 °C, and the catalyst was held at this temperature for 2 hours. After cooling to room temperature, a mixture of CH_4_ and H_2_ (100 mL min^−1^, 20/80 v/v) was introduced into the reactor. The temperature was raised at a rate of 5 °C min^−1^ to 700 °C, and the catalyst was again maintained at this temperature for 2 hours. Subsequently, the sample was allowed to cool to room temperature, and passivation was performed using a 1% O_2_/Ar gas mixture for 8 hours.^19^

Cu/Mo_2_C-1 catalyst was prepared as follows: Cu/Mo_2_C-1 catalyst was prepared by initially employing the wetting method to load Cu(NO_3_)_2_·3H_2_O onto the MoO_3_-p1 carrier.^24^ The amount of loaded Cu was determined by Cu/Mo molar ratio for X. Cu(NO_3_)_2_·3H_2_O was dissolved in a mixture of 10 mL of deionized water and 10 mL of absolute ethanol to create a copper nitrate solution, which was then stirred for further use. The MoO_3_-p1 powder was introduced into the copper nitrate solution while stirring, and the resulting aqueous mixture was dispersed at 40 °C for 1 hour using an ultrasonic machine. Subsequently, it was dried in an oven at 105 °C for 15 hours and then ground. After vacuum drying at room temperature, the sample was calcined at 500 °C for 4 hours, followed by gradual carburization in a CH_4_/H_2_ mixture (100 mL min^−1^; 20/80 v/v). The temperature was raised to 300 °C at 5 °C min^−1^ and then to 700 °C at 1 °C min^−1^, with the sample carburized at 700 °C for 2 hours before being cooled to room temperature. It was later passivated using a mixture of 1% O_2_/Ar.

Cu/Mo_2_C-2 catalyst was prepared as follows: Cu/Mo_2_C-2 catalyst was prepared similarly to Cu/Mo_2_C-1, utilizing the wetting method to load Cu(NO_3_)_2_·3H_2_O onto the MoO_3_-p2 carrier. The subsequent carburization process remained the same.

Cu/MoO_2_-1 and Cu/MoO_2_-2 catalysts were prepared as follows: for Cu/MoO_2_-1, Cu(NO_3_)_2_·3H_2_O was loaded onto the MoO_3_-p1 support using the initial wet impregnation method, excluding the carburization step. Similarly, Cu/MoO_2_-2 was prepared with Cu(NO_3_)_2_·3H_2_O loaded onto the MoO_3_-p2 support *via* initial wet impregnation without carburization.

**Fig. 1:**
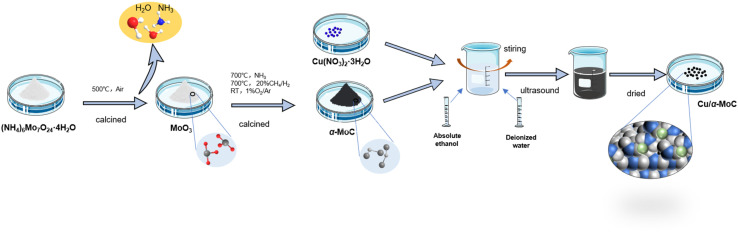
Preparation process of Cu/α-MoC catalysts.

Cu/α-MoC catalyst was prepared as follows: the Cu/α-MoC catalyst was created using the wetting method to load Cu(NO_3_)_2_·3H_2_O onto the α-MoC carrier. After drying in an oven, the sample was ground to a particle size of 40–50 mesh for later use. Preparation process of Cu/α-MoC catalyst is shown in Fig. 1. In experiments where Cu/α-MoC exhibited reduced stability, it was denoted as Cu/α-MoC*.

For comparative analysis, a CuZnAl catalyst was employed in this investigation. The catalyst, sourced from Chengdu ShengLi Technology Co. Ltd, featured a molar ratio of Cu : Zn : Al = 1 : 1 : 2. Prior to commencing the catalytic activity assessment, the Cu-based catalyst underwent a reduction process. This reduction process entailed exposure to a H_2_/Ar mixed gas flow (150 mL min^−1^; 10/90 v/v) at 250 °C for a duration of 2 hours.

### 2.2 Apparatus and tests

The MSR experiment utilized a fixed-bed stainless steel reactor from Shanghai Laibei Science Instrument Co. Ltd 2 g of catalysts, with a particle size of 250–600 μm (30–60 mesh), were loaded into the central region of the reactor and securely wrapped with fine salmon cotton to prevent catalyst dislodgment.^25^ This arrangement ensured a continuous and stable flow of gas through the catalyst bed. Prior to the experiment, approximately 2 g of passivated Cu/MoC catalyst underwent activation at 590 °C for 2 h in a CH_4_ and H_2_ mixture (15/85 v/v). Following successful activation, the sample within the reactor was cooled to the desired reaction temperature. Afterwards, the reactor was purged with flowing N_2_ gas (flow rate: 150 mL min^−1^) for a duration of 30 minutes to eliminate residual gases and establish a clean starting environment for the subsequent reaction. A mixture of H_2_O and CH_3_OH in a 3 : 1 ratio was supplied to the evaporator, which operated at 250 °C and was controlled by a feed pump at a flow rate of 0.18 mL min^−1^. The resulting steam mixture, serving as the reactant, was introduced into the reactor along with the N_2_ flow, which acted as an internal standard substance for quantifying the gas component concentrations. During the course of the reaction, the gas mixture leaving the reactor was condensed using appropriate pipes to capture any unreacted CH_3_OH and H_2_O. The collected product was subsequently subject to deoiling and drying treatments. The gas composition of the product was continuously analyzed and recorded using a real-time gas detection analyzer, specifically the Gas Board-3100 P (Hubei Cubic Ruiyi Instrument Co. Ltd). This analyzer is capable of measuring the gas concentrations of up to six components, including CO (0–20 vol%), CO_2_ (0–20 vol%), CH_4_ (0–20 vol%), C_n_H_m_ (0–5 vol%), H_2_ (0–75 vol%), and O_2_ (0–25 vol%). The measurement techniques employed were as follows: CO, CO_2_, and CH_4_ were analyzed using a non-color scattered dual-beam infrared method; H_2_ was detected using electromagnetic detection technology based on microelectronics and mechanical systems; and O_2_ was measured using electrochemical methods. The accuracy of each measurement system was validated with fully standard representations of (CO/CO_2_/CH_4_/C_*n*_H_*m*_) 1% and (O_2_/H_2_) 2%.

### 2.3 Characterization methods

After undergoing vacuum degassing, N_2_ physical adsorption was performed at a temperature of −196 °C to determine the specific surface area (*S*_BET_), pore volume (*V*_p_), and pore size (*D*_p_) of the Cu/MoC catalyst. The surface structure and interior microstructures were examined using field emission scanning electron microscopy (FESEM) and transmission electron microscopy (TEM). Furthermore, the surface chemical state of the Cu/MoC catalyst was investigated using X-ray photoelectron spectroscopy (XPS).

### 2.4 Data analysis

In the context of the MSR reaction, the primary reaction products include H_2_, CO, CO_2_, and CH_4_. To evaluate the catalytic activity of the MSR reaction, a real-time gas detection analyzer was employed to measure the concentrations of H_2_, CO, CO_2_, and CH_4_. This analysis relied on an internal standard gas and associated equations for calibration purposes. The calibration process involved generating an internal standard curve, which was established by analyzing reference gas samples with varying compositions of H_2_, N_2_, CO_2_, CO, and CH_4_.

Eqn (1) through eqn (4) were employed to determine the CH_3_OH conversion rate and the selectivity of the resulting products, namely, CO, CO_2_, and CH_4_. The molar flow rates of the reactants and products, denoted as *F*_CH_3_OH,in_, *F*_CO,out_, *F*_CO_2_,out_, and *F*_CH_4_,out_, were quantitatively expressed.1
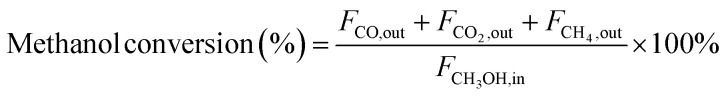
2
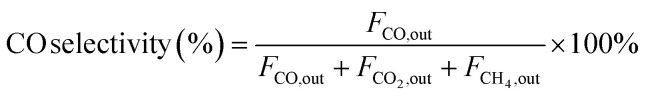
3
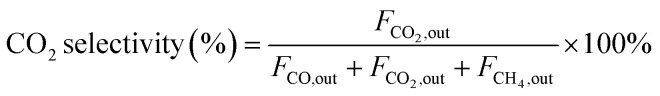
4
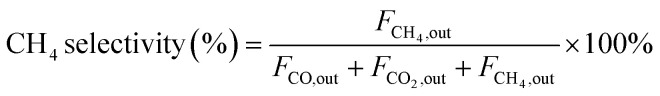


## 3 Results and discussion

### 3.1 Catalyst structure analysis

**Fig. 2:**
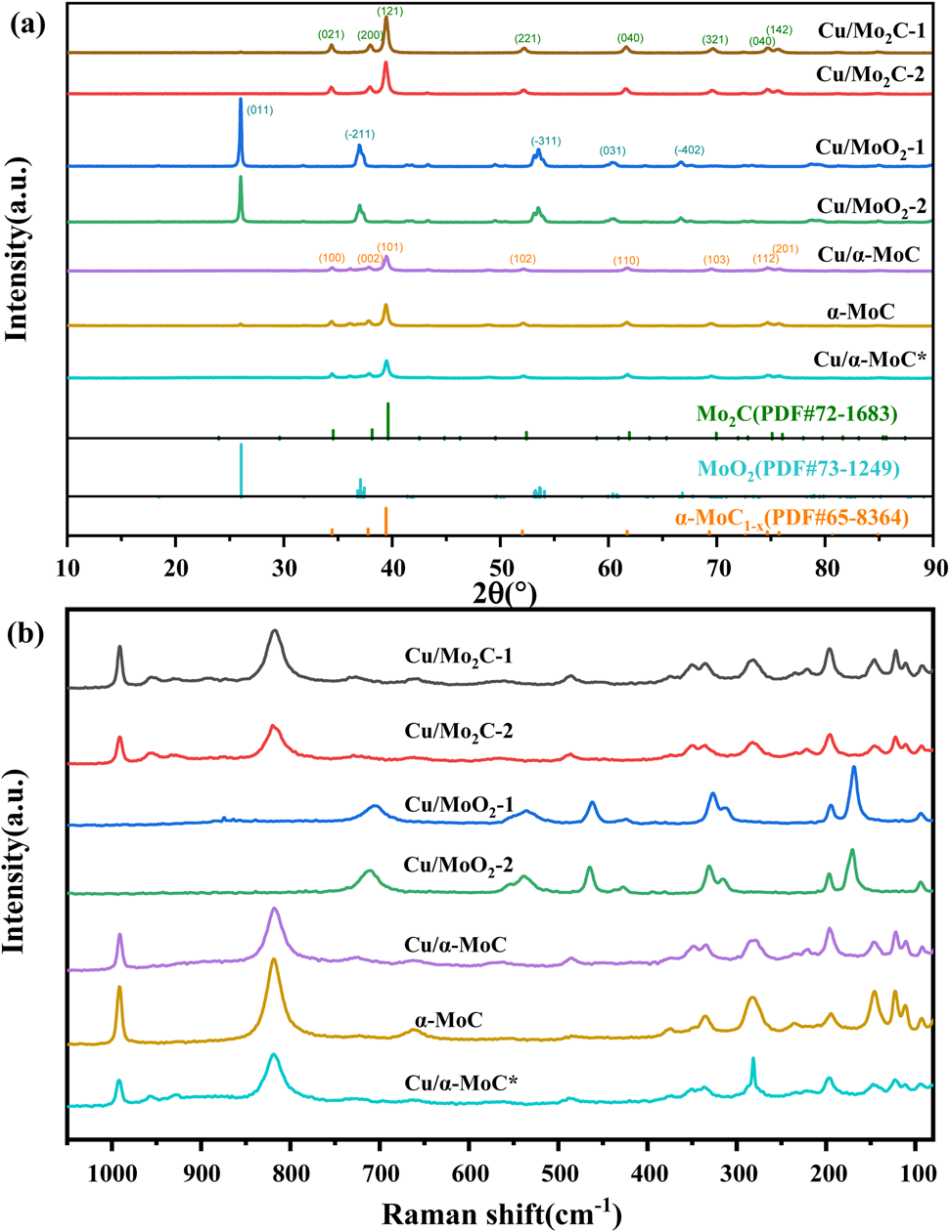
(a) XRD patterns and (b) Raman spectra of different Cu/MoC catalysts.

X-ray diffraction (XRD) analysis was conducted to elucidate the compositional characteristics of the Cu/MoC catalyst, as depicted in Fig. 2. In order to facilitate comparisons, we also included data on the well-established α-MoC catalyst and deactivated Cu/α-MoC* catalysts. The XRD patterns for Cu/Mo_2_C-1 and Cu/Mo_2_C-2 catalysts revealed prominent diffraction peaks at 34.5°, 38.1°, 39.5°, 52.3°, 61.8°, 69.8°, and 74.9° (PDF#72-1683),^26^ indicative of the Mo_2_C phase formation. Conversely, Cu/MoO_2_-1 and Cu/MoO_2_-2 catalysts did not undergo carburization at 700 °C, signifying the restoration of MoO_3_ to MoO_2_ in a methane-hydrogen hybrid atmosphere at 590 °C. Distinct peaks were observed at 26.0°, 36.9°, 37.3°, 53.0°, 53.1°, and 66.6° (PDF#73-1249).^27^ Prior to analysis, Cu/α-MoC, α-MoC, and Cu/α-MoC* catalysts underwent NH_3_ pretreatment, and their XRD patterns exhibited pronounced diffraction peaks at 34.3°, 37.6°, 39.3°, 51.9°, 61.5°, 69.1°, and 74.5° (PDF#65-8364),^28^ attributed to the α-MoC_1−*x*_ phase. Notably, no characteristic peaks corresponding to metallic Cu, Cu_2_O, or CuO were observed in any of the Cu/MoC catalysts prepared through the various synthesis methods, underscoring the homogeneous distribution of Cu species across the carburized surface.

Consistent conclusions were drawn from the Raman spectroscopic analysis. The Raman spectra of Cu/Mo_2_C-1 and Cu/Mo_2_C-2 catalysts prominently featured peaks at 77.8, 92.5, 110.8, 121.8, 145.6, 196.5, 221.9, 283.3, 335.3, 374.5, 661.6, 818.6, and 991.1 cm^−1^, unequivocally ascribed to the Mo_2_C phase (see Fig. 2b). Conversely, Cu/MoO_2_-1 and Cu/MoO_2_-2 catalysts, which remained uncarburized at 700 °C, exhibited distinct Raman peaks centered at 169.3, 194.7, 312.0, 326.3, 461.3, 535.0, and 704.6 cm^−1^. The Raman spectra of Cu/α-MoC, α-MoC, and Cu/α-MoC* catalysts consistently displayed pronounced peaks at 80.0, 95.4, 112.7, 126.1, 147.1, 196.8, 235.1, 284.5, 378.7, 663.7, 822.7, and 994.9 cm^−1^, unequivocally attributed to the presence of the α-MoC_1−*x*_ phase. When compared to catalysts containing α-MoC_1−*x*_ phases, it was observed that the primary Raman peak intensities (284.9, 822.7, and 994.9 cm^−1^) of metal-loaded catalysts experienced a significant reduction. Notably, the Raman peak intensity of the Cu/α-MoC* catalyst decreased further after the stability experiment, suggesting that the diminishment in Cu/MoC catalyst performance may be attributed to the weakening of the α-MoC_1−*x*_ phase, which subsequently led to a decrease in its catalytic activity.

N_2_ physisorption analysis was employed to characterize the physical properties of Cu/MoC catalysts, encompassing BET surface area, pore volume, and pore size distribution (see Fig. S2†). All Cu/MoC catalysts, with Cu loadings ranging from 9.82 to 14.97 wt% as outlined in Table 1, exhibited an H1 hysteresis loop. Cu/Mo_2_C and Cu/MoO_2_ catalysts presented typical type III N_2_ adsorption–desorption isotherms, whereas Cu/α-MoC catalysts displayed characteristic type IV N_2_ adsorption–desorption isotherms. The pore size distribution of Cu/MoC catalysts was primarily concentrated within the range of 10–20 nm, indicative of a mesoporous pore structure. Following carburization, Cu/Mo_2_C-1 and Cu/Mo_2_C-2 catalysts demonstrated increased BET surface areas, pore volumes, and pore sizes compared to Cu/MoO_2_-1 and Cu/MoO_2_-2 catalysts, attributable to the formation of the Mo_2_C phase during carburization. Notably, owing to the superior structural attributes of the α-MoC_1−*x*_ phase, Cu/α-MoC catalysts exhibited larger BET surface areas, reduced pore volumes, and increased pore sizes in comparison to Cu/Mo_2_C-1 catalyst. The presence of the α-MoC_1−*x*_ phase in α-MoC catalysts was corroborated by its elevated diffraction peak intensity at 39.3°. Interestingly, Cu/α-MoC* catalysts displayed an increased BET surface area, albeit with a decrease in diffraction peak intensity for the α-MoC_1−*x*_ phase (at 39.3°). This observation aligns with our earlier speculation based on Raman spectroscopy results, suggesting that the reduction in the strength of the α-MoC_1−*x*_ phase contributes to a decline in catalytic activity.

**Table 1:** Physicochemical properties of different Cu/MoC catalysts

Catalyst	*S*_BET_ a (m^2^ g^−1^)	*V*_p_ a (cm^3^ g^−1^)	*D*_p_ a (nm)	Cu content^b^ (wt%)	Mo content^b^ (wt%)
Cu/Mo_2_C-1	17.05	0.093	21.88	14.97	32.89
Cu/Mo_2_C-2	19.52	0.095	19.45	11.92	34.21
Cu/MoO_2_-1	7.81	0.030	15.41	11.52	34.00
Cu/MoO_2_-2	5.48	0.022	15.99	11.20	33.69
Cu/α-MoC	20.03	0.057	11.46	9.82	37.72
α-MoC	25.42	0.069	10.79	—	43.66
Cu/α-MoC*	29.54	0.074	9.96	7.67	32.31

a Measured by BET.

b Estimated by XPS.

**Fig. 3:**
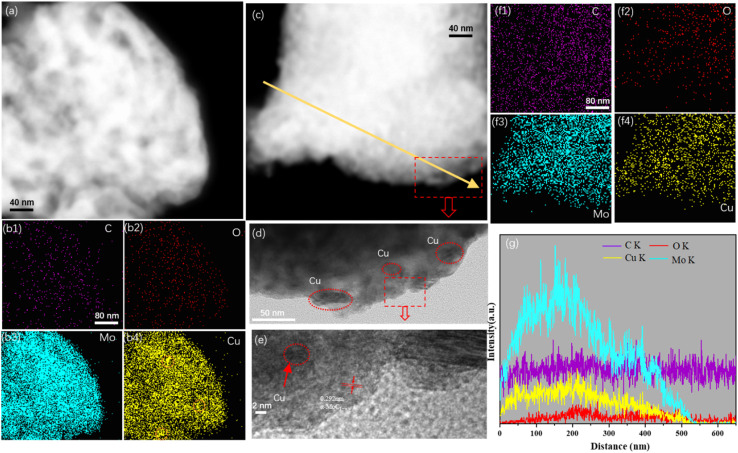
(a) TEM image and (b1–b4) corresponding EDX element mappings of Cu/Mo_2_C-1 catalyst; (c–f) TEM image and (f1–f4) corresponding EDX element mappings of Cu/α-MoC catalyst, (g) EDX line spectra along the yellow arrow in (c).

FESEM analysis was conducted to investigate the surface morphology of the Cu/MoC catalyst. The FESEM images revealed the presence of numerous small sheet-like particles and smaller particles within the Cu/α-MoC catalyst. This observation can be attributed to the sheet-like structures of varying sizes and thicknesses present in the α-MoC_1−*x*_ phase (refer to Fig. S3†). Additionally, the MoO_3_ component exhibited a platelet-like structure.^29^ Notably, due to the coexistence of particles of similar characteristics in certain regions, aggregations of particulate matter were also observed. This phenomenon may be associated with topological structural transformations occurring during the carburization of Cu. Furthermore, the microstructure of the Cu/MoC catalyst was subjected to examination through energy-dispersive X-ray (EDX) and TEM techniques, as illustrated in Fig. 3. In the case of the Cu/Mo_2_C-1 catalyst, Cu particles or clusters were uniformly dispersed across the surface of the molybdenum carbide support. Conversely, Cu particles or clusters within the Cu/α-MoC catalyst were predominantly localized on the inner surface of the molybdenum carbide support. This distribution was further validated through EDX elemental mapping analysis. These findings strongly indicate that α-MoC, as a highly active metal support, facilitates superior metal dispersion. Significantly, Cu/α-MoC catalysts exhibited a multitude of small Cu particles intricately embedded within the carbonized molybdenum phase, featuring particle dimensions ranging from 3 to 6 nm. Alongside the subnanometre Cu_*n*_ clusters and nanometre-size Cu_1_ species, nanometer-sized Cu_1_ particles were also discernible (Fig. 3d). This superior dispersion observed in the current (Cu_1_–Cu_*n*_)/α-MoC catalysts can be ascribed to the refined preparation method, which generates a greater number of surface sites for immobilizing low-dimensional Cu species.^19,29^ Consequently, the α-MoC_1−*x*_ phase displayed heightened dispersibility towards active metal species. It is noteworthy that the retention of metal particles within the molybdenum carbide bulk phase plays a pivotal role in achieving superior metal dispersion.

Fig. 3 illustrates the presence of conspicuous Cu particles, denoted by orange circles, primarily located on the inner surface of the Cu/Mo_2_C-1 catalyst. This observation, substantiated by EDX element mapping, signifies the agglomeration of Cu particles within the catalyst structure. Notably, Cu/MoO_2_-1 catalysts also exhibited the presence of relatively large Cu particles, as confirmed by the line spectrum (Fig. S4†). In stark contrast, Cu/α-MoC and Cu/α-MoC* catalysts displayed an absence of the high-dispersibility Cu_n_ clusters and Cu_1_ species, indicating the effective prevention of extensive sintering among active copper species (Fig. 3f and S5f†). Consequently, the incorporation of the α-MoC_1−*x*_ phase contributed to enhanced Cu dispersion, leading to the formation of a more efficient catalyst.^30^

**Fig. 4:**
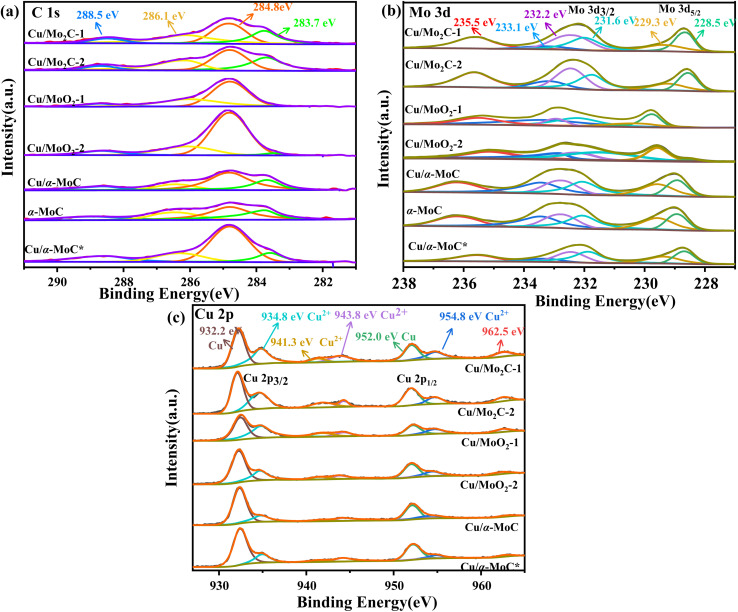
(a) C 1s, (b) Mo 3d, and (c) Cu 2p XPS spectra of different Cu/MoC catalysts.

The surface chemical composition of the Cu/MoC catalyst was thoroughly investigated using XPS, employing qualitative peak fitting to identify elemental species. The C 1s spectrum of the Cu/MoC catalyst was deconvoluted into four distinct peaks, centered approximately at 283.7, 284.8, 286.1, and 288.5 eV (Fig. 4a). The 283.7 eV peak was unequivocally assigned to carbon in the carbide form, a finding corroborated by XRD analysis, which verified the presence of the Mo_2_C phase in Cu/Mo_2_C-1 and Cu/Mo_2_C-2 catalysts.^31,32^ In contrast, Cu/α-MoC, Cu/α-MoC*, and α-MoC catalysts featured the α-MoC_1−*x*_ phase. The major and minor peaks at 284.8 and 288.5 eV, respectively, suggested the existence of non-uniform carbon species. Additionally, small peaks at 286.7 eV were attributed to carbon within C–O or C–O groups, likely formed due to passivation processes.^33^ Comparing the XPS analyses of Cu/α-MoC and Cu/α-MoC* catalysts, it became evident that the 284.8 eV peak intensity in Cu/α-MoC* catalyst was notably higher than that in Cu/α-MoC catalyst. This observation may be associated with the decreased catalytic activity of the Cu/α-MoC* catalyst, possibly stemming from the formation of carbon species within C–O or C–O groups.^34,35^

The primary peak at 283.7 eV displayed minimal variation, suggesting the relative stability of the α-MoC_1−*x*_ carbide phase throughout the experimental duration. The Mo 3d spectra of the Cu/MoC catalyst were meticulously deconvoluted, revealing three pairs of distinctive peaks, as depicted in Fig. 4b. Peaks situated around 228.5 and 231.6 eV were attributed to the Mo^2+^ species found in both the α-MoC_1−*x*_ and Mo_2_C phases.^36^ Concurrently, two additional pairs of peaks at approximately 229.3/232.2 eV (Mo 3d_5/2_) and 233.1/235.5 eV (Mo 3d_3/2_) indicated the coexistence of Mo^4+^ and Mo^6+^ species, respectively.^22^ Notably, the higher oxidation state of Mo observed in the Cu/MoC catalyst resulted from the inevitable surface oxidation.

In Fig. 4c, the Cu/MoC catalyst exhibited a dense split spin-orbital composition peak in the Cu 2p spectrum, appearing at approximately 933 and 952.75 eV, which posed challenges in unequivocally distinguishing the chemical state of Cu solely *via* XPS.^37^ Specifically, two discernible peaks at 932.2 and 934.8 eV were derived from the Cu 2p_3/2_ signal, corresponding to Cu^0^ and Cu^+^, respectively. It is worth noting that the Cu 2p_3/2_ signal was somewhat superimposed with Cu^2+^, indicating the potential existence of Cu in multiple chemical states, a possibility that cannot be entirely ruled out. However, the characteristic satellite structure associated with Cu^2+^ at approximately 943 and 962.5 eV typically exhibits strong peaks,^38,39^ implying the presence of CuO in the Cu/MoC catalyst. When compared to Cu/Mo_2_C-1 and Cu/α-MoC catalysts, the intensified peaks at 943 and 962.5 eV were evidently attributed to the presence of the α-MoC_1−*x*_ phase, thereby negating the formation of Cu^2+^.

### 3.2 MSR study

#### 3.2.1. Catalytic activity test

**Fig. 5:**
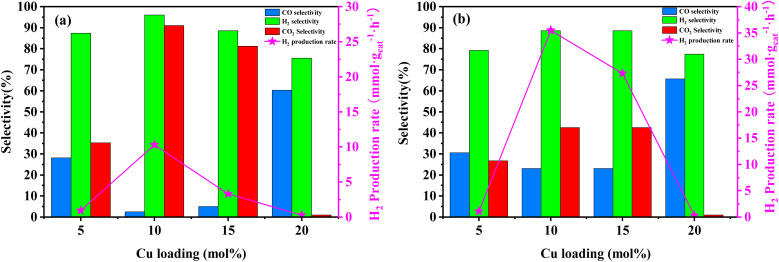
Activity results for MSR performed on Cu/Mo_2_C-1 catalyst at different loads of copper. (a) Reactor temperature at 250 °C; (b) reactor temperature at 350 °C. Experimental conditions: methanol flow rate, 0.2 mL h^−1^; nitrogen flow rate, 150.0 mL min^−1^; catalyst loading, 2 g; reactor pressure, 1 bar; data acquisition carried out from 0.5 to 2.5 h of running time.

Activity experiments were conducted on Cu/Mo_2_C-1 catalysts with varying Cu contents at two different temperatures, 250 and 350 °C (see Fig. 5). At 250 °C, as the Cu content in Cu/Mo_2_C-1 increased from 5 to 10 mol%, there was a significant enhancement in the rate of H_2_ production, ranging from 1 to 10 mmol g_cat_^−1^ h^−1^. Moreover, the selectivity for H_2_ increased from 87.4% to 96.1%, while the selectivity for CO_2_ increased from 35.3% to 91.1%. This behavior can be attributed to the improved dispersion of active metal particles of Cu/MoC catalyst on the carbonized molybdenum surface, leading to superior catalytic activity.^21^ However, as the Cu content further increased from 10 to 20 mol% in Cu/Mo_2_C-1 catalyst, the H_2_ generation rate gradually declined, nearly approaching 0 mmol g_cat_^−1^ h^−1^. Additionally, the selectivity for H_2_ and CO_2_ gradually decreased, while the selectivity for CO increased. This drop in catalytic activity can be attributed to excessive loading of active metal particles, resulting in their agglomeration and consequent loss of catalytic activity.^22^ It's noteworthy that the H_2_ generation rate of the 10 mol% Cu/Mo_2_C-1 catalyst followed a normal distribution pattern. This particular catalyst composition exhibited exceptional catalytic activity with minimal aggregation of active metal Cu particles, demonstrating remarkable hydrogen yield and methanol conversion.

Table 2 compiles an overview of the research outcomes regarding the performance of diverse copper-based catalysts in MSR over recent years. The findings highlight that the Cu/α-MoC catalyst demonstrates outstanding hydrogen production capabilities coupled with reduced selectivity towards CO. The XRD analysis reveals the presence of the α-MoC_1−*x*_ phase in the Cu/α-MoC catalyst, a characteristic that enhances the rate of hydrogen generation (Fig. 2a). Further insights from TEM images and EDX element mapping elucidate superior copper dispersibility within the Cu/α-MoC catalyst, contributing to an augmented rate of hydrogen generation (Fig. 3a and b). Comparative analysis with recent studies on copper-based catalysts suggests that loading copper onto α-MoC effectively mitigates CO production during formaldehyde decomposition, a phenomenon attributed to the distinctive α-MoC_1−*x*_ phase. Therefore, Cu/α-MoC catalyst has a greater potential for commercial development.

**Table 2:** Selection of Cu-based catalysts for MSR reported in recent years

Catalyst	*T* (°C)	*n*H_2_O : *n*CH_3_OH	H_2_ production rate (mmol_H_2__ g_cat_^−1^ h^−1^)	CO/CO_2_ selectivity (%)	H_2_ selectivity (%)	References
Cu/Mo_*x*_C_*y*_	300	1	60	∼2.8/∼20 (mol%)	∼67 (mol%)	29
Cu/ZrSi30	260	1.3	38	—	—	39
CuO–Al_2_O_3_	450	5	—	∼2.5/-	∼85	40
Cu/ZnAl-LDHs/γ-Al_2_O_3_	300	1.2	7.5	1/-	—	41
CuZnO/Al_2_O_3_	260	1	1.4	0.1/-	—	42
Cu/Mo_2_C-1	350	3	37.05	2.85/46.84	60.5	This work
Cu/α-MoC	350	3	44.07	0.10/69.52	79.8	This work

The catalytic performance of various Cu/MoC catalysts in MSR was assessed at 350 °C (Table 3). Cu was incorporated into the α-MoC catalyst, resulting in Cu/α-MoC catalyst, which exhibited a notable increase in methanol conversion rate and H_2_ yield. This underscores the substantial influence of introducing Cu species into MoC, significantly enhancing the MSR response.^29^ Comparative analysis of catalysts containing three phases, namely Mo_2_C, MoO_2_, and α-MoC_1−*x*_, was carried out using XRD results. Cu/Mo_2_C-1 catalyst displayed an H_2_ yield of 37.05 mmol g_cat_^−1^ h^−1^ and a methanol conversion of 42.39%. Notably, the carbonization of the MoO_3_-1 precursor, prepared from ammonium molybdenum, was highly effective.^20^ Confirmation of the carbon peak in the catalyst was achieved *via* Raman spectroscopy (Fig. 2). In contrast, the catalyst containing the MoO_2_ phase exhibited a significantly lower H_2_ production rate of only 19.02 mmol g_cat_^−1^ h^−1^, even lower than that of the unloaded α-MoC catalyst, indicating poor MSR catalytic performance of MoO_2_. In contrast, Cu/α-MoC catalyst demonstrated the highest H_2_ yield, reaching 44.07 mmol g_cat_^−1^ h^−1^, with an average turnover frequency (ATOF) of 28.5 mol mol_Cu_^−1^ h^−1^ and an impressive H_2_ selectivity of 78.9%. Notably, the Cu/α-MoC catalyst, featuring the α-MoC_1−*x*_ phase, exhibited a larger S_BET_ and smaller *D*_P_ compared to other Cu/MoC catalysts. Consequently, among the Cu/MoC catalysts tested, Cu/α-MoC with the α-MoC_1−*x*_ phase demonstrated superior MSR response and hydrogen production efficiency.^23^

**Table 3:** Catalytic activity results for MSR performed on different Cu/MoC catalysts^a^

Catalyst	Metal loading (wt%)	Convension (%)	ATOF (mol_H_2__ mol_Cu_^−1^ h^−1^)	H_2_ production rate (mmol_H_2__ g_cat_^−1^ h^−1^)	CO selectivity (%)	CO_2_ selectivity (%)
α-MoC	—	22.87	—	19.73	3.19	49.41
Cu/Mo_2_C-1	14.97	42.39	15.72	37.05	2.85	46.84
Cu/Mo_2_C-2	11.92	21.62	14.11	26.49	2.98	42.21
Cu/MoO_2_-1	11.52	14.08	9.18	16.65	7.09	41.76
Cu/MoO_2_-2	11.20	34.99	10.78	19.02	2.81	61.62
Cu/α-MoC	9.82	33.38	28.50	44.07	0.10	69.52
CuZnAl	—	39.48	—	38.00	15.05	83.17

a Experimental conditions: methanol flow rate, 12 mL h^−1^; nitrogen flow rate, 150.0 mL min^−1^; catalyst loading, 2 g; reactor temperature, 350 °C; reactor pressure, 1 bar; data acquisition carried out from 0.5 to 2.5 h of running time.

**Fig. 6:**
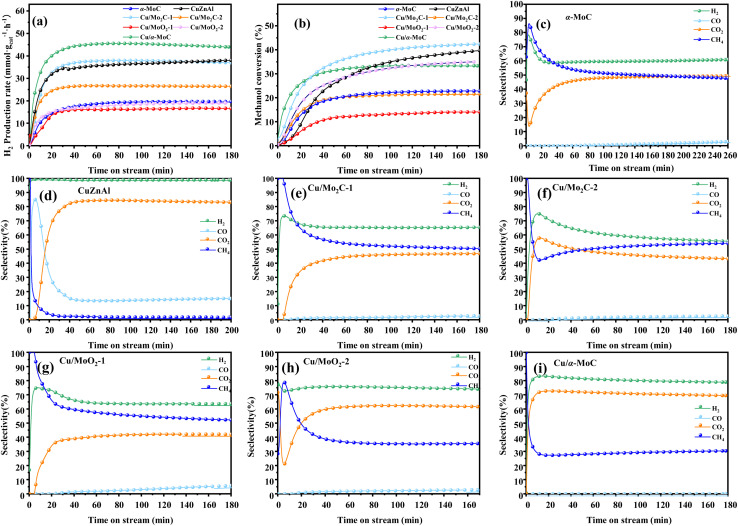
Stability test for MSR performed on different MoC-based catalysts at 350 °C: (a) H_2_ production rate, (b) methanol conversion, and (c–i) product selectivity. For experimental conditions, refer to Table 3.

For further comparison, the catalytic activity of a commercial catalyst for methanol reforming, CuZnAl, was tested to evaluate the performance of Cu/MoC catalysts. As indicated in Table 2, the CuZnAl catalyst achieved a hydrogen yield of 38 mmol g_cat_^−1^ h^−1^, albeit with significantly high CO selectivity (15.05%). In Fig. 6, the commercial CuZnAl catalyst exhibited high selectivity for H_2_ and CO_2_ but also displayed substantial CO selectivity. In contrast, Cu/α-MoC catalyst demonstrated superior selectivity for H_2_ and CO_2_ compared to other Cu/MoC catalysts, while maintaining exceptionally low CO selectivity. This resulted in the production of more hydrogen than the commercial CuZnAl catalysts.

**Fig. 7:**
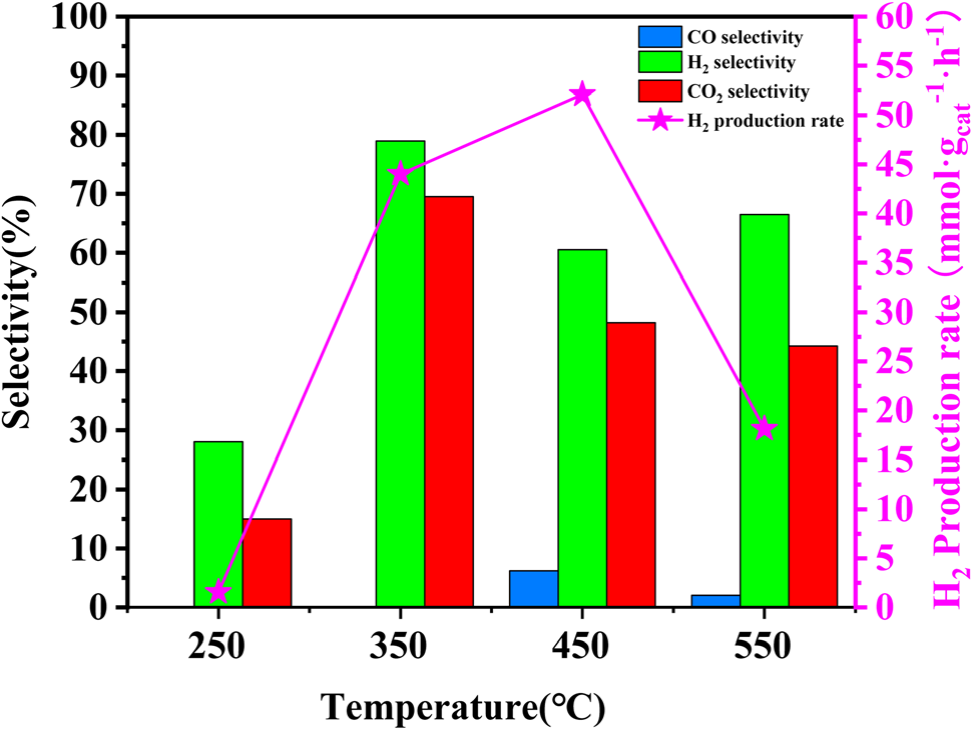
Activity results for MSR performed on Cu/α-MoC catalyst at different temperatures. Experimental conditions: methanol flow rate, 0.2 mL h^−1^; nitrogen flow rate, 150.0 mL min^−1^; catalyst loading, 2 g; reactor pressure, 1 bar; data acquisition carried out from 0.5 to 2.5 h of running time.

In order to assess the catalytic activity of MSR over a relatively stable 2.5 hours period, experiments were conducted at a range of reaction temperatures (250, 350, 450, and 550 °C) (Fig. 7). The Cu/MoC catalyst was loaded with 10% based on its molecular weight. At 300 °C, the H_2_ yield reached 44.07 mmol g_cat_^−1^ h^−1^, with H_2_ and CO_2_ selectivities of 79.8% and 69.5%, respectively. Subsequently, raising the temperature to 450 °C led to an 8.03 mmol g_cat_^−1^ h^−1^ increase in H_2_ yield, achieving 52.10 mmol g_cat_^−1^ h^−1^. However, the selectivity for H_2_ decreased to 60.52%, while the selectivity for CO increased to 6.18%. At 450 °C, the likelihood of a side reaction generating CO (HCHO → CO + H_2_) became evident.^43^ Further elevating the temperature to 550 °C resulted in a reduction of H_2_ yield to 18.1 mmol g_cat_^−1^ h^−1^. It was observed that higher temperatures caused catalyst sintering, leading to carbon deposition. Simultaneously, the α-MoC_1−*x*_ phase underwent oxidation at elevated temperatures, reverting to Mo_2_C, thereby diminishing the H_2_ yield.^21,25^ Upon evaluating the hydrogen production rate and methanol conversion rate, it was determined that the optimal temperature for MSR over the Cu/α-MoC catalyst was 350 °C.

#### 3.2.2. Stability test

**Fig. 8:**
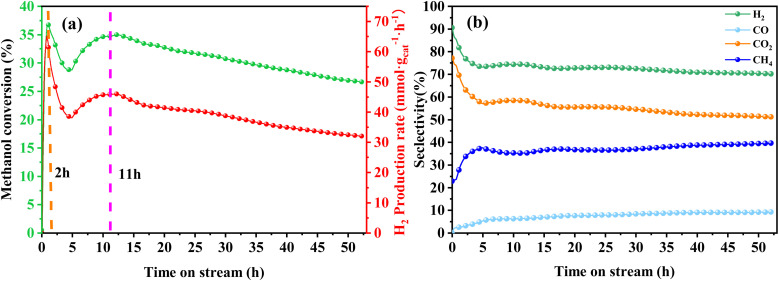
Stability test for MSR performed on Cu/α-MoC catalyst at 350 °C: (a) methanol conversion and H_2_ production rate, (b) product selectivity. For experimental conditions, refer to Table 3.

The extended-term stability of the Cu/α-MoC catalyst in the context of MSR was assessed at 350 °C, a temperature of practical significance, with a feeding rate set at 12 mL h^−1^. In the initial 2 hours of operation, the Cu/α-MoC catalyst underwent an activation phase marked by exceptionally high catalytic activity, as illustrated in Fig. 8a. During this period, methanol conversion rates reached nearly 37%, yielding an impressive 60 mmol g_cat_^−1^ h^−1^ of H_2_. Following this activation phase, an 11 hours transition period ensued, during which the methanol conversion rate gradually declined by 35%, with a corresponding reduction in H_2_ yield to 43 mmol g_cat_^−1^ h^−1^. This modest catalyst deactivation was observed.

A comprehensive analysis of the Cu/α-MoC and Cu/α-MoC* catalysts presented in Table 1 revealed a decrease in the weight percentage of Cu within the Cu/α-MoC catalyst, dropping from 9.82 to 7.67 wt%. Furthermore, there was a reduction in pore diameter, indicative of changes in the molecular configuration of Cu. XPS analysis of the Cu 2p_3/2_ signal demonstrated an increase in the peak area corresponding to Cu^2+^ at 932.6 eV in the Cu/α-MoC* catalyst with prolonged reaction time, signaling the gradual oxidation of Cu to CuO. Remarkably, the H_2_ selectivity remained relatively stable as the reaction progressed, consistently hovering around 75% (Fig. 8b). Concurrently, as the reaction time increased, the selectivity of CH_4_ and CO exhibited an upward trend, while the selectivity of CO_2_ witnessed a decrease.

Furthermore, the intensity of the diffraction peak associated with the α-MoC_1−*x*_ phase (at 39.3°) in the Cu/α-MoC* catalyst decreased over time. This variation in the strength of the α-MoC_1−*x*_ phase significantly contributed to the observed decline in catalytic activity. Impressively, even after 55 hours of continuous operation, the Cu/α-MoC* catalyst still exhibited well-dispersed Cu, with only a small quantity of Cu particles accumulating on the MoC surface, a fact confirmed through TEM and EDX element mapping (Fig. S7†). This observation suggests that the strong interaction between Cu particles and the α-MoC_1−*x*_ phase might be responsible for the noteworthy Cu dispersion on the Cu/α-MoC* catalyst.^22,29^

#### 3.2.3. Proposed reaction pathway

Numerous investigations have delved into unraveling the intricate reaction mechanism underlying the behavior of carbonized molybdenum catalysts during MSR. In this catalytic process, methanol undergoes a series of transformations, giving rise to various products such as CO, CO_2_, and CH_4_.^44^ Despite extensive research efforts, the precise reaction mechanism governing carbonized molybdenum catalysts in MSR has remained elusive. Controversy persists regarding the pathway leading to the reorganization of methanol steam into H_2_ and CO_2_. Of significance are the intermediates methyl formate and formaldehyde, considered pivotal in the MSR process.^45,46^ These intermediates follow distinct conversion pathways; for instance, CH_3_COOH, originating from methanol, desorbs into the gas phase and subsequently decomposes into CH_4_ and CO_2_.

5 2CH_3_OH ⇌ HCOOCH_3_ + 2H_2_, Δ*H* = +4 kJ mol^−1^

6 HCOOCH_3_ ⇌ CO_2_ + CH_4_, *ΔH* = −71 kJ mol^−1^

7 HCOOCH_3_ + H_2_O ⇌ CH_3_OH + HCOOH, Δ*H* = +14 kJ mol^−1^

8 HCOOH ⇌ CO_2_ + H_2_, Δ*H* = +31 kJ mol^−1^

9 CH_3_OH ⇌ HCHO + H_2_, Δ*H* = +130 kJ mol^−1^

10 HCHO ⇌ CO + H_2_, Δ*H* = −2 kJ mol^−1^

Drawing upon existing literature and experimental product analysis, this study ventures to elucidate the reaction pathway for methanol steam reformation and hydrogenation. The primary reactions are depicted in eqn (5)–(10). As portrayed in Fig. 8b, the selectivity towards CO and CH_4_ escalates with reaction time, indicative of CH_3_COOH decomposition into CO_2_ and CH_4_ (eqn (6)), and HCHO decomposition into CO and H_2_ (eqn (10)). As elucidated earlier, HCHO and CH_3_COOH are recognized as pivotal intermediates in MSR. Consequently, CH3OH decomposition into CH_3_COOH and H_2_ (eqn (5)) and CH3OH decomposition into HCHO and H_2_ (eqn (9)) must also be present. In Fig. 6i, the Cu/α-MoC catalyst exhibits remarkable H_2_ and CO_2_ selectivity. The Cu active sites within the Cu/α-MoC catalyst foster the generation of substantial quantities of CO_2_ and H_2_ through HCOOH decomposition (eqn (8)). In accordance with pertinent research on MoC, a significant presence of methoxy groups is detected at the interface of the α-MoC_1−*x*_ site, leading to the formation of CH_3_COOH, while a minor fraction of CH_3_COOH is hydrolyzed into CH_3_OH and HCOOH (eqn (7)). Eventually, HCOOH undergoes decomposition, yielding CO_2_ and H_2_.

**Fig. 9:**
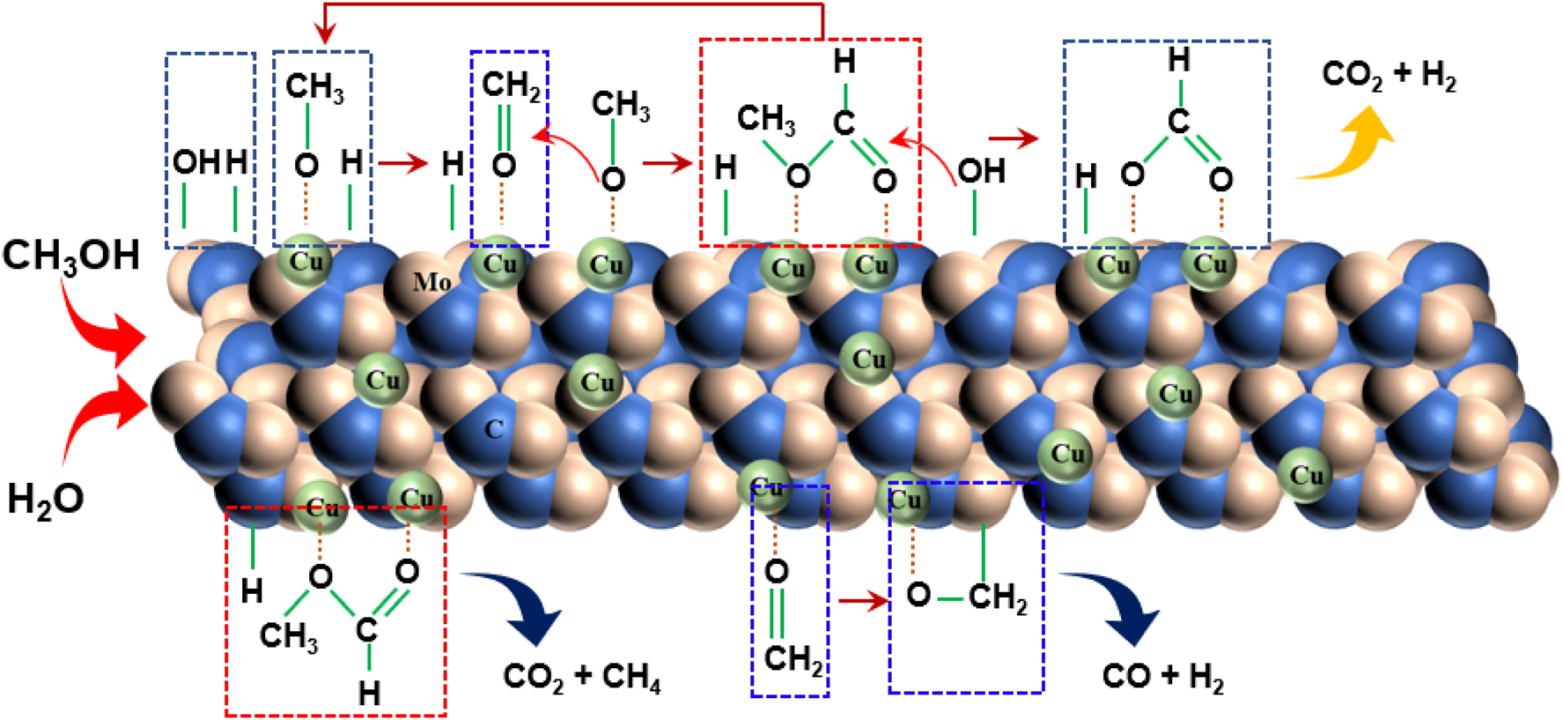
Proposed reaction pathway for MSR performed on Cu/α-MoC catalyst.

Drawing on the literature and our experimental findings, we propose the principal pathways governing the MSR reaction on the Cu/α-MoC catalyst, while acknowledging the potential existence of additional reactions. As depicted in Fig. 9, the upper segment illustrates the primary reaction pathway, commencing with the decomposition of methanol into a methoxyl group, followed by further dehydrogenation to produce aldehyde at the α-MoC_1−*x*_ site interface. The resulting aldehydes subsequently interact with the methyl group to generate the intermediate methyl formate. Hydrolysis of methyl formate yields formic acid, which is subsequently transformed into carbon dioxide and hydrogen gas. The lower segment encompasses certain side reactions, wherein methane and carbon dioxide are directly decomposed by methyl formate, leading to the production of carbon monoxide and hydrogen during formaldehyde breakdown.

## 4 Conclusion

In the course of this investigation, we synthesized diverse Cu/MoC catalyst variants and evaluated their performance in the context of MSR. As part of our approach, we leveraged various characterization techniques to underscore the feasibility of modulating the MoC phase's composition by controlling the catalyst preparation method. At an operational temperature of 350 °C, the Cu/α-MoC catalyst exhibited a remarkable carbon monoxide selectivity of zero, while concurrently achieving a hydrogen selectivity nearing 80%. Furthermore, it demonstrated a noteworthy hydrogen production rate of 44.07 mmol g_cat_^−1^ h^−1^, surpassing both the commercial CuZnAl catalyst (38 mmol g_cat_^−1^ h^−1^) and the Cu/Mo_2_C-1 catalyst (37.05 mmol g_cat_^−1^ h^−1^). Of paramount significance is the presence of highly dispersed Cu particles within Cu/α-MoC, with an average size of approximately 4 nm. This dispersion phenomenon arises from the robust interaction between the subnanometre Cu_n_ clusters, nanometre-size Cu_1_ species, and α-MoC_1−*x*_ phases. Nevertheless, following a continuous 50 hours operational period at 350 °C, the catalytic activity of the Cu/α-MoC catalyst for hydrogen production *via* MSR displayed a decline. This observed reduction can be ascribed to structural changes occurring within the α-MoC_1−*x*_ phase throughout the reaction process. Upon an amalgamation of activity and stability assessments, it is evident that Cu/α-MoC catalysts hold significant promise, especially given their heightened stability. These findings lay the groundwork for advancing the utilization of carbonized molybdenum catalysts in the realm of MSR.

## Author contributions

Wen Jiang: writing – review & editing, writing – original draft, visualization, validation, investigation, formal analysis, data curation. Aonan Liu: validation, software. Ming Yao: supervision, methodology. Yuchun Zhang: supervision, methodology. Peng Fu: validation, software, resources, project administration, methodology, investigation, funding acquisition, conceptualization.

## Conflicts of interest

There are no conflicts to declare.

## Supplementary Material

RA-014-D3RA07448J-s001
