# Supplementary material for: Achieving efficient almost CO-free hydrogen production from methanol steam reforming on Cu modified α-MoC
Source: RSC Adv. 2024 Jan 10;14(3):2036–47. doi: 10.1039/d3ra07448j (PMC10777472; doi:10.1039/d3ra07448j)
Supplement: RA-014-D3RA07448J-s001 [file RA-014-D3RA07448J-s001.pdf]

Electronic Supplementary Material (ESI) for RSC Advances.
This journal is © The Royal Society of Chemistry 2023

Achieving efficient almost CO-free hydrogen production from methanol by anchoring Cu_1-Cu_n to α-MoC to enhance their strong interaction

Wen Jiang[a], Aonan Liu[a], Ming Yao[a], Yuchun Zhang[a]*, Peng Fu[a]*

[a] School of Agricultural Engineering and Food Science, Shandong University of Technology, Zibo255000, China†

Email address: zhangyc@sdut.edu.cn (Y. Zhang), fupengsdut@163.com (P. Fu).

* Corresponding author. *Email address:* zhangyc@sdut.edu.cn (Y. Zhang), fupengsdut@163.com (P. Fu).

Characterization Methods

The specific surface area (S_{BET}) and pore volume (V_p) of Cu/MoC catalysts were measured by N_2 physisorption at −196 °C in a BSD-PS2 physical adsorption and desorption apparatus from Bayside Instruments, China. Before the measurement, the test sample was degassed under high vacuum at 200 °C for 6 hours to remove the moisture from the surface and pores.

The phase compositions of Cu/MoC catalysts were analyzed by using the power X-ray diffraction technique (XRD) with the SHIMADZU XRD-6100 diffractometer (SHIMADZU, Japan) using Cu Kα as radiation at 40 kV and 30 mA. The data for XRD patterns of the test samples were collected in the range of 2θ = 10-90° with a scanning rate of $4°\cdot min^{-1}$. The acquired diffraction patterns of the samples were analyzed by MDI Jade 5.0 and compared to those of standard database.

The Raman spectra of Cu/MoC catalysts were recorded at room temperature by using a laser confocal Raman spectrometer (LabRAM HR Evolution, HORIBA JobinYvon, France) with 532 nm laser excitation. During the measurement, the laser beam was focused on the test sample by adjusting the location of sample platform through the laser safety goggles, and the scanning range was from 80 to 1300 cm^{-1}.

The surface morphologies of Cu/MoC catalysts were measured by transmission electron microscopy (TEM, FEI Tecnai G2F 20, USA) at a low accelerating voltage of 5 kV. Before the measurement, a small quantity of test sample was uniformly attached to conductive carbon tape on a round aluminum slab.

The surface morphologies of Cu/MoC catalysts were measured using a field emission scanning electron microscope (FESEM, FEl Quanta 250,USA). This electron microscope was equipped with

an energy dispersive spectrometer. In a typical measurement, a small amount of test sample was thoroughly mixed with anhydrous ethanol by using ultrasonic dispersion (100 kHz) for half an hour, and a drop or two of the suspension was dripped on a holey nickel grid coated with an amorphous carbon film.

The surface chemical state of Cu/MoC catalysts was measured using a Thermo ESCALAB 250Xi X-ray multifunctional imaging electron spectrometer with an Al Kα radiation source. The binding energies of all elements in the catalysts were calibrated by using the C1s peak at 284.8 eV from neutral carbon as the reference.

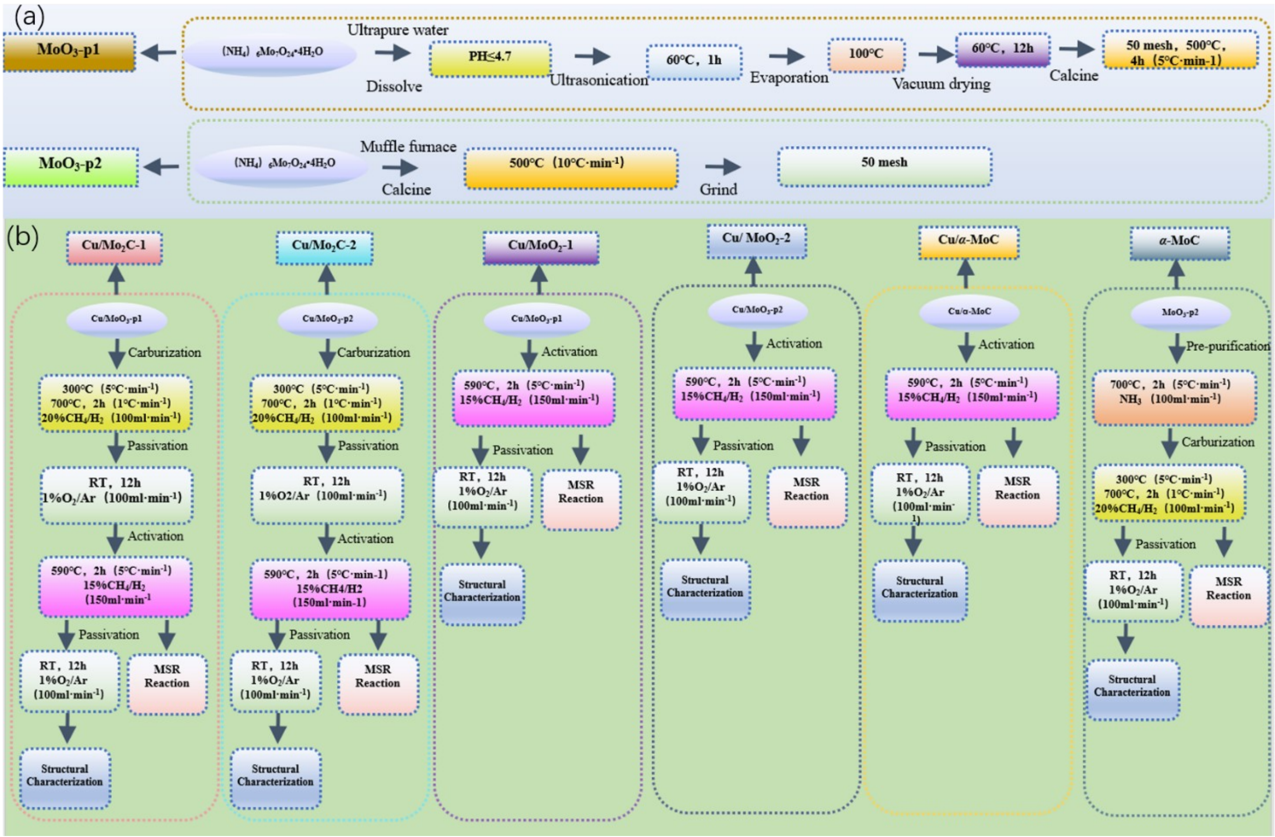

Fig. S1. (a) Schematic diagram of the preparation process of different MoO_3 supports and (b) Schematic diagram of the preparation process of different Cu/MoC catalysts

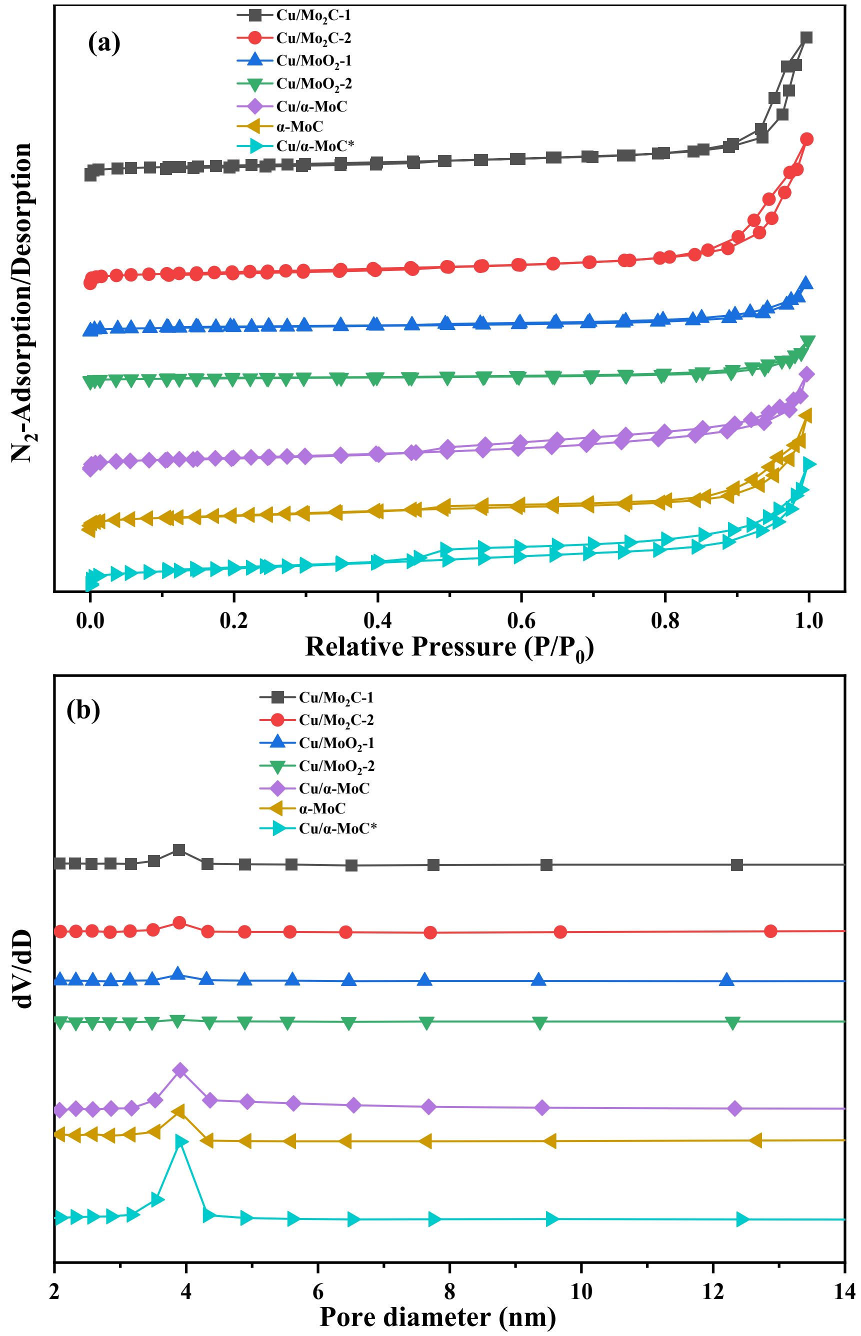

Figure S2. (a) Nitrogen adsorption-desorption isotherms and (b) BJH pore size distribution profiles of different Cu/MoC catalysts.

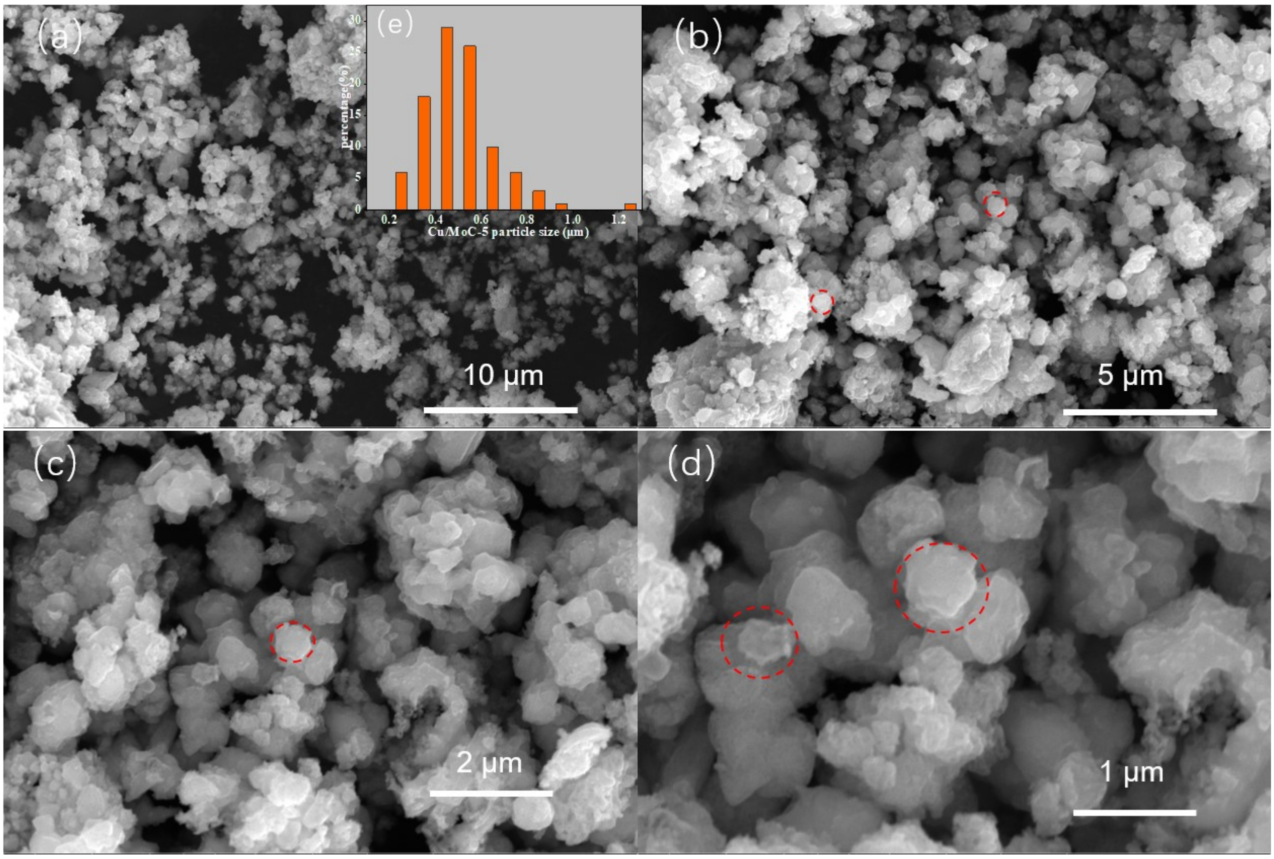

Figure S3. (a-d) FESEM images of Cu/MoC-5 catalysts, (e) MoC particle size distribution of Cu/α-MoC catalyst.

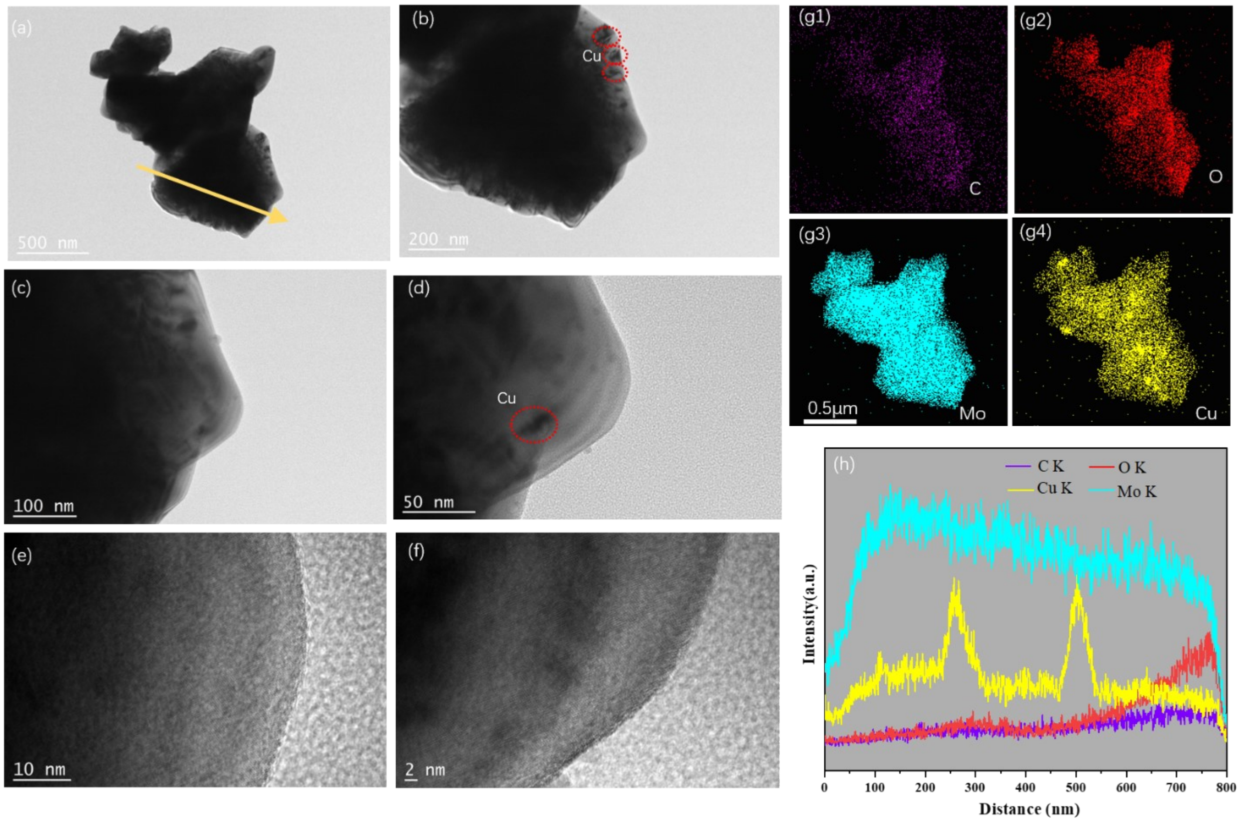

Figure S4. (a-f) TEM image and (g1-g4) corresponding EDX element mappings of Cu/MoO_2-1, (e) EDX line spectra along the yellow arrow in (a).

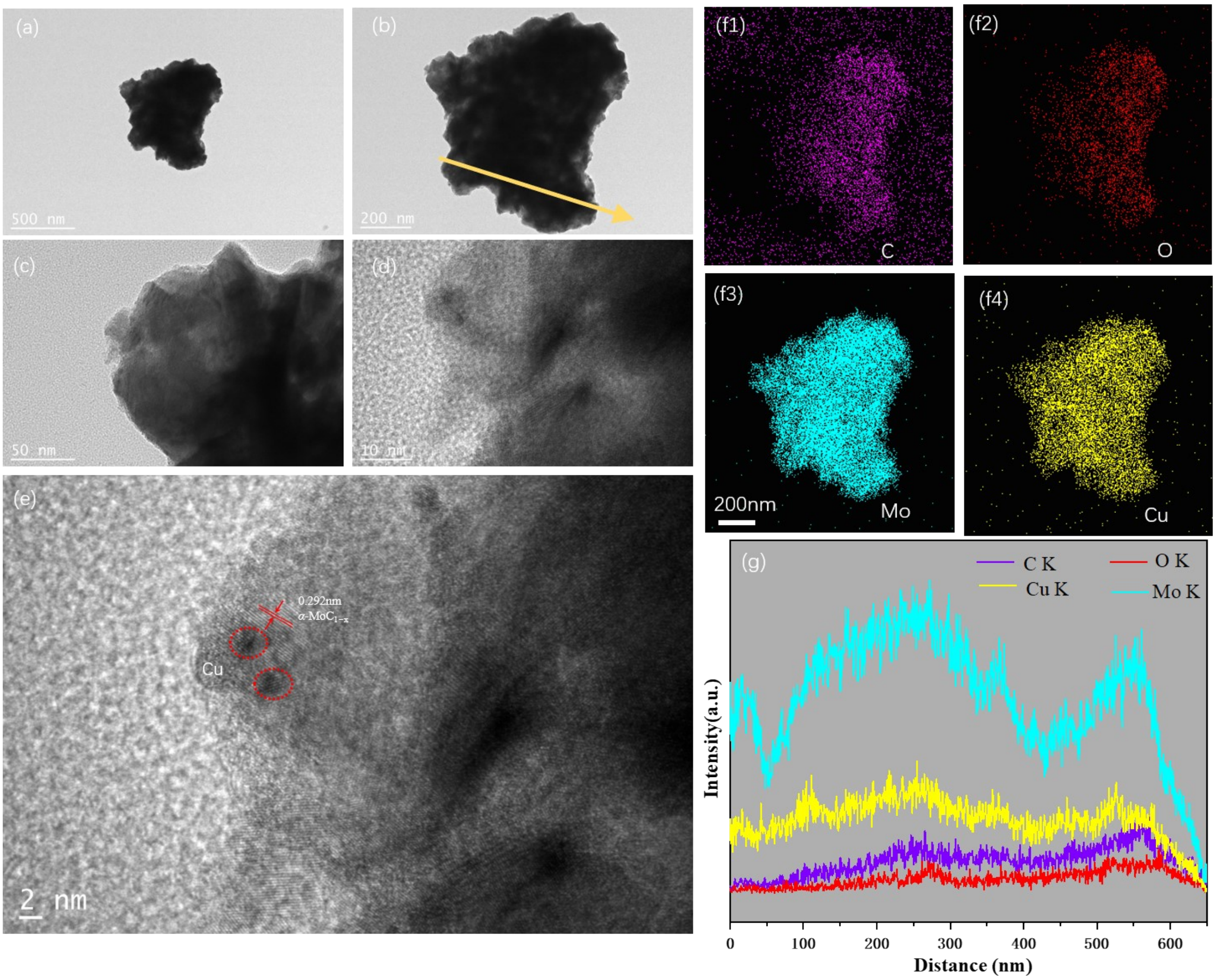

Figure S5. (a-e) TEM image and (f1-f4) corresponding EDX element mappings of Cu/α-MoC*, (e) EDX line spectra along the yellow arrow in (b).
